# Topical Delivery of Dual Loaded Nano-Transfersomes Mediated Chemo-Photodynamic Therapy against Melanoma via Inducing Cell Cycle Arrest and Apoptosis

**DOI:** 10.3390/ijms25179611

**Published:** 2024-09-05

**Authors:** Yiping Guo, Wenxiao Zhong, Cheng Peng, Li Guo

**Affiliations:** 1 State Key Laboratory of Southwestern Chinese Medicine Resources, Chengdu University of Traditional Chinese Medicine, Chengdu 611137, China; yguo8@ucmerced.edu; 2 School of Pharmacy, Chengdu University of Traditional Chinese Medicine, Chengdu 611137, China; zhongwenxiao@stu.cdutcm.edu.cn

**Keywords:** melanoma, chemo-photodynamic therapy, cell cycle, apoptosis

## Abstract

Melanoma is a malignant skin cancer associated with high mortality rates and drug resistance, posing a significant threat to human health. The combination of chemotherapy and photodynamic therapy (PDT) represents a promising strategy to enhance antitumor efficacy through synergistic anti-cancer effects. Topical delivery of chemotherapeutic drugs and photosensitizers (PS) offers a non-invasive and safe way to treat melanoma. However, the effectiveness of these treatments is often hindered by challenges such as limited skin permeability and instability of the PS. In this study, transfersomes (TFS) were designed to facilitate transdermal delivery of the chemotherapeutic drug 5-Fluorouracil (5-FU) and the PS Imperatorin (IMP) for combined chemo-photodynamic therapy for melanoma. The cytotoxic and phototoxic effects of TFS-mediated PDT (TFS-UVA) were investigated in A375 cells and nude mice. The study also demonstrated that TFS-UVA generated intracellular ROS, induced G2/ M phase cell cycle arrest, and promoted cell apoptosis. In conclusion, this study indicated that 5-FU/ IMP-TFS serves as an effective transdermal therapeutic strategy for chemo-PDT in treating melanoma.

## 1. Introduction

Melanoma originates from abnormal melanocytes in the basal layer of the epidermis and has experienced a significant increase in recent years, with an estimated 99,700 new cases of melanoma expected in the United State in the year of 2024 [1,2,3]. Although surgical excision remains the primary treatment for early-stage melanoma, it can result in severe side effects such as large skin defects [4]. Melanoma is known for its resistance to conventional treatments including chemotherapy, radiotherapy, as well as innovative treatments such as immunochemotherapy and bio-chemotherapy [5,6,7]. Therefore, it is necessary to develop more effective therapeutic strategies to fill the gap for better melanoma treatments.

5-Fluorouracil (5-FU) is a chemotherapeutic agent known for its broad-spectrum anti-cancer activities against colorectal, breast, and skin cancers. The therapeutic mechanism involves cell apoptosis by blocking thymidylate synthase, thereby disrupting DNA synthesis [8]. However, systemic delivery of 5-FU via oral and intravenous routes can lead to gastrointestinal and cardiac toxicities [9]. To solve this issue, transdermal delivery of 5-FU has been introduced to restrict systemic toxicities and enhance local drug concentration. However, with poor skin permeability and low local absorption, the applications of 5-FU were limited by its hydrophilic nature [10]. The root of *Angelica dahurica* (Chinese name: Baizhi), has been used in Traditional Chinese Medicine to treat skin diseases for over 1700 years [11]. Imperatorin (IMP), the primary active component found in *Angelica dahurica*, exhibits strong pharmacological effects such as anti-cancer and anti-inflammatory properties [12]. Researchers have demonstrated its concentration-dependent inhibition manner and synergistic effects with cisplatin against melanoma growth [13,14]. In the murine model, IMP administered intraperitoneally under UVA illumination showed significant inhibition of melanoma cell viability [15]. This suggests that IMP could serve as a novel Photodynamic Therapy (PDT) photosensitizer for treating melanoma. Chemo-PDT represents a promising approach to overcoming the limitations of single-treatment modalities and enhancing total treatment efficiency [16]. PDT is a safe and effective non-invasive treatment used in dermatological conditions. Topical PDT involves the application of PS to the skin and specific wavelengths of light for the generation of reactive oxygen species to induce cell apoptosis through oxidative damage [17]. PDT is widely used for skin diseases such as psoriasis [18], vitiligo [19], squamous cell carcinoma of the skin, and melanoma [20,21]. However, challenges remain as the effectiveness of PDT is often hindered by skin penetration and PS instability [22].

Transfersomes (TFS) are a type of liposome characterized by their highly elastic vesicles [23]. Composed of phospholipids and edge-activating agents (EA), TFS destabilize the lipid bilayer to increase vesicle deformability [24,25]. Compared to liposomes, TFS have a smaller particle size and more elastic liposomes, enabling them to deform and squeeze through the interstitial spaces of stratum corneum cells without compromising their structures [26]. This property enhances its skin permeability and local drug concentration during topical administration [27,28]. Additionally, TFS share a similar phospholipid bilayer structure with liposomes, which improves drug solubility and stability. The vesicle structure enhances the effective encapsulation of both hydrophilic and hydrophobic drugs [29]. TFS has shown its benefits in delivering drugs to localized lesions for the treatment of melanoma [30].

In this study, we focused on developing an efficient chemo-PDT using TFS as a nanodrug carrier for treating melanoma. The optimized TFS formulation was determined using the Box-Behnken-Design (BBD) and characterized by particle size, potential, Fourier transform infrared spectroscopy, and transmission electron microscopy (TEM). Drug release kinetics were evaluated using the dialysis method and skin permeation properties were assessed using the Franz diffusion cell method in vitro, respectively. The therapeutic efficacy of chemo-PDT was evaluated through both in vitro and in vivo experiments. We additionally explored the effects of photocytotoxicity on the cell cycle and cell apoptosis in A375 cells. These results signify that this transdermal drug delivery system can be a probable chemo-PDT candidate for the treatment of melanoma.

## 2. Results

### 2.1. The BBD Studies

**Figure 1:**
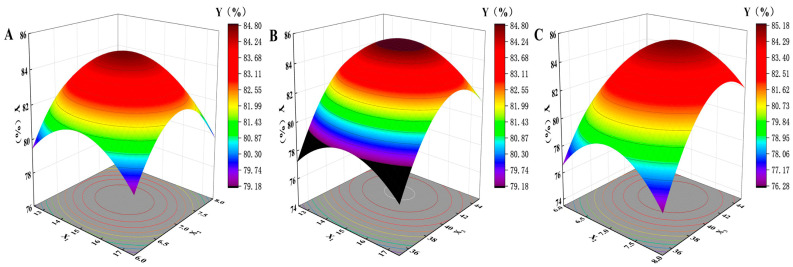
3D response surface plot of encapsulation efficiency. (**A**) Drug/lipid ratio and SPC/TW-80 ratio; (**B**) drug/lipid ratio and hydration temperature; (**C**) SPC/TW-80 ratio and hydration temperature.

The BBD optimization strategy was developed with three main factors: drug/lipid ratio (*X*1), the SPC/TW-80 ratio (*X*2), and the hydration temperature (*X*3), where the co-encapsulation efficiency (*Y*) was set as the experimental response value. Three different levels of the factors are defined in Table 1, and the 3D response surface plot is shown in Figure 1. Optimization data can be found in Supplementary Materials: Tables S1 and S2. The results were analyzed by ANOVA using Design-Expert 8 software (R^2^ = 0.9795), and *Y* was calculated using the following equation:

(1) $$\begin{array}{l} Y=0.85-2.96\times 10^{-3}\times X1+4.412\times 10^{-3}\times X2+0.022\times X3-1.702\times 10^{-3}\times X1X2\\ \;\;\;\;\;-3.143\times 10^{-3}\times X1X3+5.623\times 10^{-3}\times X2X3-0.021\times {X1}^{2}-0.029\times {X2}^{2}\\ \;\;\;\;\;-0.032\times {X3}^{2} \end{array}$$

### 2.2. TFS Preparation, Characterization and Optimization

**Figure 2:**
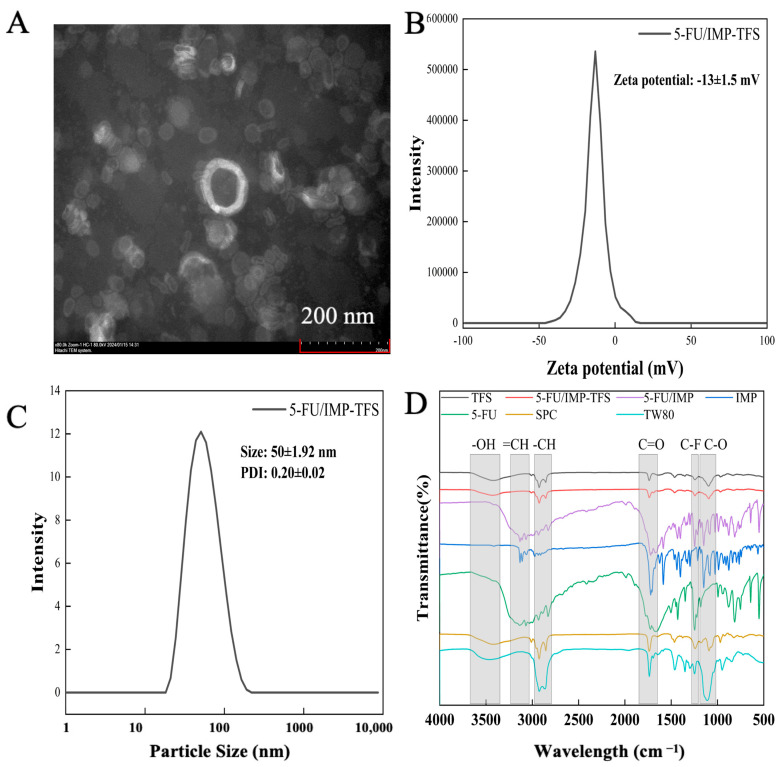
Characterization of TFS. (**A**) TEM image of 5-FU/IMP-TFS; (**B**) the zeta potential of TFS; (**C**) the particle size distribution of TFS; (**D**) the FT-IR spectroscopy of each group.

Studies have shown that lipid vesicles with smaller particle sizes enhance the penetration of encapsulated drugs into the skin [31]. The optimized formulation of 5-FU/IMP-TFS had a smaller particle size of 50 ± 1.92 nm (Figure 2C) and a uniform size distribution with a PDI value of 0.20 ± 0.02 (less than 0.3). Vesicle size reduction of TFS may be due to the addition of surfactants and using a probe sonicator. Zeta potential is a predictor of formulation stability. High zeta potential values reduce aggregation by electrostatic interactions and enhance formulation stability. The optimized zeta potential of 5-FU/IMP-TFS was −13 ± 1.5 mV (Figure 2B), which is consistent with the results reported by Afzal Hussain [32]. The HPLC analysis revealed that the loadings of 5-FU and IMP were 2.49 ± 0.02% and 2.81 ± 0.07%, respectively. The encapsulation efficiency of 5-FU and IMP in the formulations were 79.71 ± 0.65% and 89.89 ± 2.16%, respectively. These results indicate that TFS has a small particle size and good dispersibility, making it capable of forming a stable co-delivery system for hydrophilic (5-FU) and hydrophobic drugs (IMP). The methodology for HPLC analysis is shown in Figures S1 and S2, and Tables S3–S8.

TEM revealed the microstructure of 5-FU/IMP-TFS (Figure 2A). With 80 kV accelerating voltage, 80 k magnification, and a work distance of 11.8 mm, the TEM image presented the membranes of 5-FU/IMP-TFS vesicles, with a clearly visible phospholipid bilayer and a smooth, structurally intact surface. Due to its bilayer membrane structure, TFS can co-deliver lipophilic and hydrophilic drugs [33]. The FTIR spectra (Figure 2D) confirmed the successful preparation of TFS. The characteristic absorption peaks of IMP were observed at 3106 cm^−1^ (=CH), 1718 cm^−1^ (C=O), and 1145 cm^−1^ (C-O), while those of 5-FU were observed at 3045 cm^−1^ (=CH), 1650 cm^−1^ (C=O), and 1251 cm^−1^ (C-F). The physical mixture of 5-FU and IMP exhibited a superposition of characteristic peaks. This suggested successful encapsulation of 5-FU and IMP in the TFS.

### 2.3. In Vitro Drug Release and Ex Vivo Skin Permeation Studies

**Figure 3:**
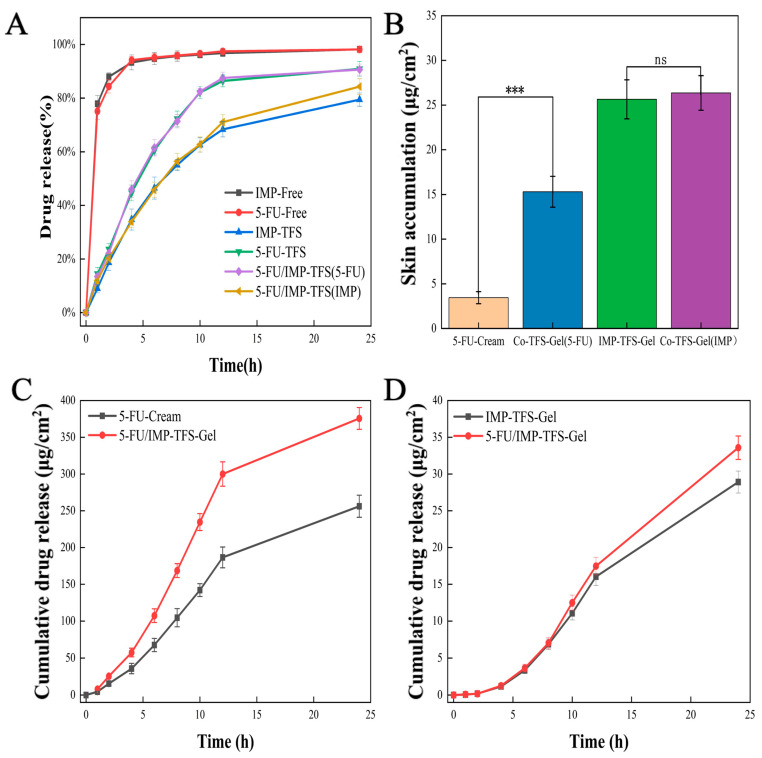
Drug release studies, skin permeation, and deposition studies. (**A**) The drug release profiles. (**B**) Drug deposited into the skin after treatment with different formulations. (**C**) Rat skin permeation profiles of 5-FU loaded formulations. (**D**) Rat skin permeation profiles of IMP loaded formulations. (*** *p* < 0.001, ns indicates statistically no significance).

This study evaluated the in vitro drug release profiles of both drugs in free form or TFS-loaded form (single-loaded or co-loaded) in a water/ethanol release medium (*v*/*v* 70:30) (Figure 3A). The free drug released around 80% of the medication within an hour, while the drug-loaded TFS gradually released 90% of 5-FU and 80% of IMP over a 24 h period. The results showed that drugs using TFS as a carrier have a certain sustained release effect compared to the free drug. There was no significant difference in the release rate between single-drug loading and co-drug loading.

TFS formulations are fluid and cannot be applied topically. Therefore, TFS was mixed with a Carbopol gel matrix for topical administration [34]. Carbopol gel is a hydrophilic gel matrix that is easily applied and absorbed into the skin and is often used as a vehicle for topical dermal administration [35]. Incorporating the TFS liquid formulation into a semi-solid gel for topical delivery can improve drug stability and patient compliance. Permeation studies were conducted using a Franz diffusion cell (Figure 3) to evaluate the quantitative percutaneous permeation of FU-Cream and TFS-gel formulations across excised rat abdominal skin. The fluxes for 5-FU were 11.71 ± 1.1 μg/cm^2^/h and 17.24 ± 2.1 μg/cm^2^/h for the FU-Cream and 5-FU/IMP-TFS-Gel, respectively. The cumulative penetration amount per unit area of FU-Cream and 5-FU/IMP-TFS-Gel in 24 h was 256.19 ± 15.00 μg/cm^2^ and 375.63 ± 14.84 μg/cm^2^, respectively (Figure 3C). For IMP, the fluxes were 1.30 ± 0.08 μg/cm^2^/h and 1.49 ± 0.09 μg/cm^2^/h for the IMP-TFS-Gel and 5-FU/IMP-TFS-Gel, respectively. The IMP-TFS-Gel and 5-FU/IMP-TFS-Gel had a cumulative penetration amount of 28.91 ± 1.50 μg/cm^2^ and 33.58 ± 1.60 μg/cm^2^ per unit area, respectively, in 24 h (Figure 3D). Following the permeation studies, the gel formulations underwent drug deposition study in the same experimental conditions. For drug deposition, FU-Cream, Co-TFS-Gel (FU), IMP-TFS-Gel, and Co-TFS-Gel (IMP) demonstrated a total skin accumulation of 3.45 ± 0.67 μg, 15.30 ± 1.73 μg, 25.65 ± 2.19 μg, and 26.36 ± 1.793 μg, respectively (Figure 3B). Specifically, the steady-state rate of 5-FU in TFS was 1.47 times greater than the cream group, and the retention amount was 4.43 times that of the cream. However, there was no significant difference for IMP. The results of drug release and the skin permeation tests indicated that TFS gel was more effective for on-site skin delivery compared to conventional creams. The stratum corneum barrier effect was reduced and skin penetration was enhanced due to the small particle size and hyper-deformability of TFS [36]. Additionally, drug deposition in the skin was improved by the slow release of TFS and the reticular structure of the gel matrix.

### 2.4. Cytotoxicity and Phototoxicity Studies In Vitro

**Figure 4:**
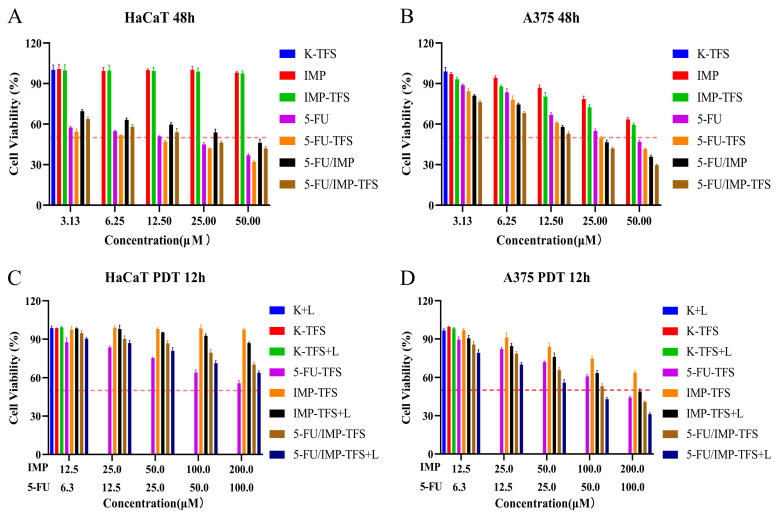
Cytotoxicity and phototoxicity. (**A**) Dark toxicity study of cell viability of HaCaT cell line for 48 h. (**B**) Dark toxicity study of cell viability of A375 cell line for 48 h. (**C**) Phototoxicity study of cell viability for HaCaT cell line for 12 h (1.5 J/cm^2^, UVA). (**D**) Phototoxicity study of cell viability of A375 cell line for 12 h (1.5 J/cm^2^, UVA). Red dash lines reflect the half maximal inhibitory concentrations of each group.

This study evaluated the effects of the cytotoxicity and phototoxicity of the drugs on HaCaT and A375 cells using the CCK-8 assay. 5-FU is known as a hydrophilic drug that faces challenges crossing the lipophilic cell membranes, resulting in low intracellular concentrations [37]. IMP is a lipophilic compound with low water solubility, poor bioavailability, and rapid degradation in cell culture media [27]. In this study, TFS was performed with anti-cancer and photosensitizing agents in a phospholipid bilayer to decrease restriction and facilitate cellular uptake. In Figure 4, results show that drug-loaded TFS formulations are more cytotoxic than the free drug. It is noteworthy that K-TFS formulations without drug loading did not reduce cell viability under any conditions, which supports the contention that safe nanocarriers can enhance cytotoxicity. Studies have proven that solar ultraviolet radiation (UVR) is a leading factor in many skin diseases [38]. However, a study conducted by Otilia Gag showed that UVA (10 J/ cm^2^) did not have a cytotoxic effect on HaCaT and A375 cells, whereas UVB (0.5 J/cm^2^) significantly reduced cell viability [39]. In addition, our studies confirmed that there was no significant toxicity in HaCaT and A375 cells under UVA (1.5 J/cm^2^) irradiation. Furthermore, the combination of UVA with furanocoumarins and their derivatives has been reported to inhibit melanoma cells [40,41].

Although 5-FU restricts DNA synthesis and inhibits cell growth, its lack of selectivity for cancer cells can harm normal cells and cause side effects such as gastrointestinal reactions and dermatitis [42]. We developed a 48-h dark toxicity study of 5-FU and IMP on normal HaCaT cells (Figure 4A). The cell survival rate of both IMP and IMP-TFS was over 95%. The half maximal inhibitory concentration (IC_50_) values of 5-FU and 5-FU/IMP on HaCaT cells were 10.92 and 35.36 μM (calculated from the 5-FU concentration), respectively. The study results indicate that IMP did not exhibit cytotoxic effects on HaCaT cells. Furthermore, the co-delivery of 5-FU and IMP resulted in a significant reduction in the cytotoxicity of 5-FU on HaCaT cells [43]. An assessment of the antitumor activity of 5-FU and IMP was conducted on melanoma A375 cells using a 48-h dark toxicity test (Figure 4B). Our study discovered that both 5-FU and IMP reduced the viability of A375 cells in a concentration-dependent manner. The IC_50_ values for 5-FU and 5-FU/IMP were 36.89 and 21.64 μM (calculated from the 5-FU concentration), respectively. Although IMP and IMP-TFS had limited antitumor effects, the combination of 5-FU and IMP significantly enhanced the antitumor effects. In summary, the combination of IMP and 5-FU reduces toxicity to normal cells and enhances the inhibitory effect on tumor cells.

Chemo-photodynamic therapy can overcome the limitations of monotherapy and improve antitumor activity [44,45]. In studies of cellular phototoxicity, the cellular activity of PDT decreased gradually as the concentration of IMP increased (Figure 4C). IMP-TFS+L damaged both normal and tumor cells, but the viability of HaCaT cells at the highest concentration of IMP-TFS+L was still above 85%, indicating biosafety. The IC_50_ for 5-FU-TFS, 5-FU/IMP-TFS, and 5-FU/IMP-TFS+L were 78.8, 60.2, and 35.24 μM (calculated from the 5-FU concentration), respectively (Figure 4D). Among these, 5-FU/IMP-TFS+L exhibited the strongest antitumor effect on A375 cells. The results indicate that PDT has biosafety and significant antitumor activity, and the combination of chemo-photodynamic therapy can further enhance the antitumor efficacy.

### 2.5. Intracellular Reactive Oxygen Species Generation

**Figure 5:**
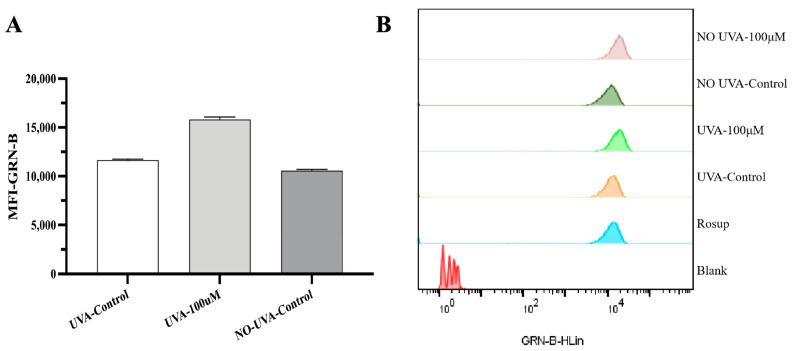
Effect of TFS/ UVA photodynamic treatments on ROS generation in A375 cells. (**A**) The quantification of intracellular ROS generated in different groups. (**B**) Cells were stained with DCFH-DA and the fluorescence intensity was measured using the flow cytometry.

In chemo-photodynamic therapy, the photosensitizers IMP can react with molecular oxygen and generate cytotoxic reactive oxygen species (ROS) which induces oxidative damage to accelerate cell death with the excitation of UVA. To verify and quantify the ROS excited from the photochemistry of 5-FU/IMP-TFS (aka. TFS) and UVA, intracellular ROS generation was measured by staining with DCFH-DA assay and analyzed by flow cytometry. As shown in Figure 5, no obvious change can be observed in ROS generation for the groups in the dark. The quantification of intracellular ROS is presented in Figure 5A. When A375 cells were exposed to UVA, the difference in ROS generation between groups was obvious, as shown in Figure 5B. The TFS-UVA group could produce much more ROS than the TFS-only group. This result indicates that increasing intracellular ROS was induced by the photodynamic activation between IMP and UVA, resulting in the combination therapeutic efficacy in treating A375 cells.

### 2.6. Cell Apoptosis Analysis

**Figure 6:**
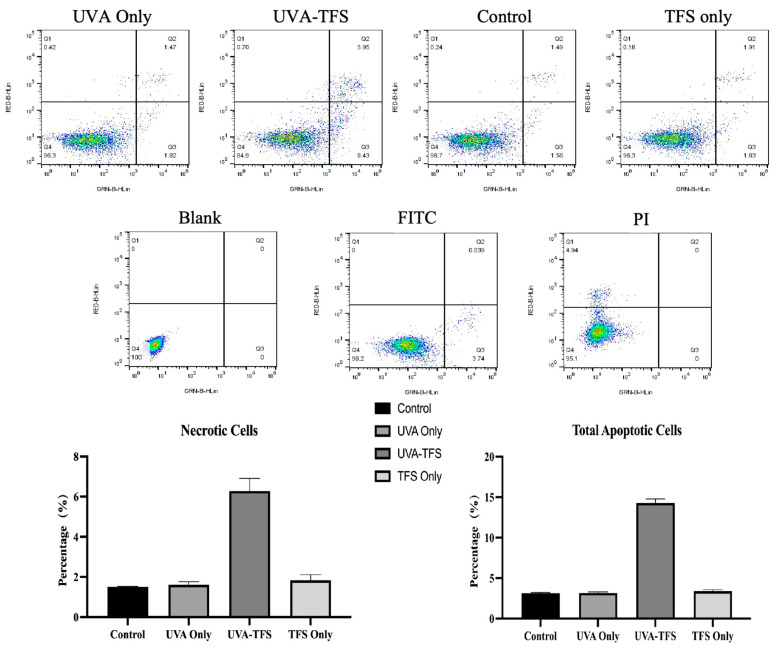
TFS/ UVA photodynamic treatments induced apoptosis in A375 cells. Cells were stained with Annexin V-FITC and PI before flow cytometric analysis.

The percentage of apoptotic A375 cells was assessed using Annexin V-fluorescein isothiocyanate (FITC) and propidium iodide (PI) double staining for flow cytometry analysis. High Annexin V and low PI staining (Q3) indicated early apoptosis of cells. in contrast, strong staining signals of both Annexin V label and PI (Q2) showed the cells were in the stage of necrosis or late apoptosis. As shown in Figure 6, 14.38% of UVA-irradiated cells were found to be apoptotic (early apoptosis plus late apoptosis) when incubated with TFS. However, the UVA-only and the drug-only groups showed no significant cell apoptosis. This assay indicated that chemo-photodynamic therapy could induce apoptosis and necrosis in A375 cells.

### 2.7. Cell Cycle Analysis

**Figure 7:**
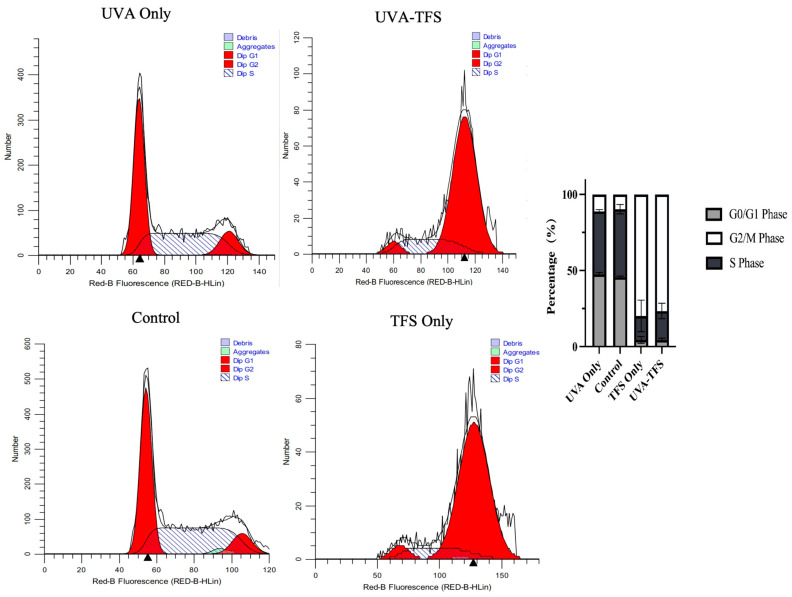
TFS/ UVA photodynamic therapy-induced cell cycle arrest of A375 cells. Cells were stained with PI and the cell cycle distribution was measured by flow cytometric analysis. (Triangles indicate the most PI-stained DNA content measured by flow cytometry).

PI was used to measure the cell DNA content. As shown in Figure 7, the treatment of UVA did not alter the cell cycle compared to the control group. Cells treated with TFS and UVA-TFS showed a significant increase in G2/M phases. These results indicated that chemo-photodynamic therapy could impact cell cycle progression and cause cell cycle arrest in the G2/M phase, leading to the inhibition of the cells.

### 2.8. Antitumor Therapeutic Efficacy In Vivo

**Figure 8:**
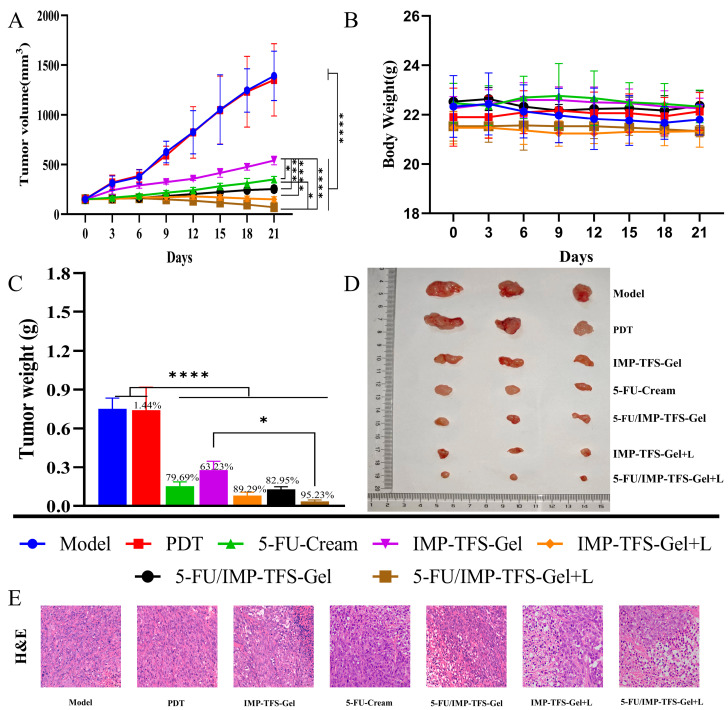
Antitumor therapeutic efficacy in vivo. (**A**) Tumor growth inhibition curves in mice with different treatment regimens (*n* = 3). (**B**) Photos of tumors. (**C**) The weight of tumors. (**D**). Body weight of mice during treatment. (**E**) H&E staining of tumor tissues captured at ×400 magnification. Data are presented as mean ± SD, *n* = 3; * *p* < 0.05, *** *p* < 0.001, **** *p* < 0.0001.

A375 tumor-bearing nude mice models were used to validate the antitumor effect of topical PDT co-administered with 5-FU and IMP. Once the tumor volume reached 150 mm^3^, the A375 tumor-bearing nude mice were randomly divided into 7 groups and treated with different protocols. All groups displayed an inhibitory effect on tumor growth except the model and PDT groups (Figure 8A). Photographs of tumor tissues treated with the different regimens are presented in Figure 8B. These results are consistent with the changes in tumor volume. The group treated with 5-FU/IMP-TFS-Gel and UVA Laser exhibited the highest tumor inhibition effect, with an IRT of 95.23% (Figure 8C). No significant weight loss was observed in any group during the treatment period. This indicated that the treatment regimens had a good biosafety profile (Figure 8D). To evaluate the antitumor effect in vivo, we conducted H&E staining and histological studies of tumor tissues. The tumor cells in the model and PDT groups were densely packed with large nuclei and active nuclear division. In contrast, the tumor cells in the treatment group exhibited necrotic cell disintegration, loss of cell membrane structure, and residual cellular debris. The level of necrosis in tumor cells varied across the different treatment groups, with the highest level of apoptosis observed in the 5-FU/IMP-TFS-Gel with UVA Laser treatment group.

## 3. Discussion

The encapsulation of drugs within the nano drug delivery systems (NDDS) has been extensively studied over the past few decades and has demonstrated great potential in cancer treatments [46,47]. Systemic administration is typically achieved through intravenous injection, providing rapid onset of action and improved drug bioavailability [48]. However, due to biological barriers, only a fraction of the therapeutic dosage from intravenous administrations reaches the tumor sites and residues [49]. Therefore, systemic administration may require high doses or multiple administrations, which can lead to side effects [50]. Administering drugs locally for skin cancer offers advantages such as non-invasive administration during long-term treatment and increased local drug concentration with minimal toxicity [16]. Additionally, local delivery of multi-functional nanoparticles for photodynamic and photothermal therapy against tumor cells could exhibit enhanced antitumor efficiency [51]. The electrostatic interaction and photochemistry of inorganic materials and ligands would contribute to tumor inhibition [52].

In this study, nano-transfersomes co-loaded with 5-FU and IMP were designed, and their transdermal delivery was further explored. TFS-gel could improve local drug concentration and skin permeability. The strongest antitumor activity was observed in 5-FU/IMP-TFS-Gel with UVA Laser, which may be attributed to several factors. TFS gel offers a promising option for NDSS, with better penetration and patient adherence to skin cancer treatments. The ability to deliver PS agents and chemotherapeutic drugs altogether to the cancerous sites shows a great potential for skin diseases. Additionally, the topical delivery system increases the local concentration of the drug at the affected site, resulting in a rise in PS concentration and enhanced topical PDT. Results have indicated that chemo-PDT significantly inhibited the growth and proliferation of melanoma. However, current studies were limited as antitumor activities were only carried out in cell lines like HaCaT and A375, but not in skin cells or organs. Notably, the evaluation of antitumor agents has not only involved human cancer cells (A375 and A431), but also the human normal skin cell lines (TE353.sk). The study showed that proapoptotic agents like saponins can induce cell apoptosis in cancer cells in a dose-dependent manner. However, they did not induce the same effect in TE353.sk normal cells [53]. Similarly, TE353.sk cells and LoVo cells were employed in a study to investigate the photokilling selectivity and efficiency of TiO_2_ nanoparticles in PDT. The results indicated that combined chemo-PDT could kill all LoVo cancer cells within 90 min, while it killed only 39% of TE353.sk normal cells [54]. The TE353.sk normal human dermal fibroblasts (NHDF) were utilized for testing the proapoptotic and prooxidative effects of gallium sensitizers compared to melanoma cancer cells and keratinocytes [55]. This study has proven that these second-generation sensitizers complemented with metal ions did not inhibit the viability of fibroblasts and keratinocytes, but significantly reduced the viability of cancer cells. This indicates that an ideal antitumor agent should provide advanced therapeutic effects in cancer cells with limited toxicity to normal cells. To validate the effectiveness of Chem-PDT agents such as 5-FU/IMP-TFS, further studies are crucial, particularly those focusing on selective targeting and evaluating cytotoxicity across normal skin cells and keratinocytes. Additionally, it would act selectively efficiently in cancer cells while being less active in normal cells. While chemo-PDT shows promise in treating melanoma, ongoing studies are needed to ensure it achieves the desired efficacy without compromising normal cells. Future research should refine these therapies to enhance selectivity and minimize toxicity, thereby providing safer and more effective strategies for cancer treatment.

## 4. Materials and Methods

### 4.1. Materials

Imperatorin (purity > 98%) was purchased from Push Bio-Technology Co., Ltd. (Chengdu, China), and 5-FU (purity > 98%) was obtained from Macklin Biochemical Co., Ltd. (Shanghai, China). Soybean lecithin was purchased from A. V. T. Pharmaceutical Co., Ltd. (Shanghai, China). Methanol (HPLC grade) was obtained from Thermo Fisher Scientific (Shanghai, China). HQ fetal bovine serum (FBS) was purchased from TransGen Biotech Co., Ltd. (Beijing, China). DMEM cell culture medium was purchased from Gibco (MA, USA). Cell Counting Kit-8 (CCK-8) was obtained from Labgic Technology Co., Ltd. (Beijing, China). Ultra-pure water was produced using an ULUPURE integral water purification system. All other chemical reagents were of analytical grade.

### 4.2. Cell Culture and Animals

Human immortalized keratinocyte cell lines, HaCaT, and human malignant melanoma cells, A375, were obtained from ATCC, cultured in DMEM with 10% FBS and 1% penicillin/streptomycin maintained at 37 °C in a 5% CO_2_ incubator. Male SD rats (200 g) and female BALB/c-Nu mice (4–5 weeks, 18–22 g) were obtained from SiPeiFu Biological Technology Co., Ltd. (Beijing, China). All animal experiments were conducted under the guidelines approved by the Institutional Animal Care and Use Committee (IACUC) of Chengdu University of Traditional Chinese Medicine.

### 4.3. Preparation of TFS

TFS encapsulating IMP and 5-FU were prepared using the thin film hydration method. Soybean lecithin (SPC), Tween-80 (TW-80), and IMP were dissolved in methanol/chloroform (*v*/*v* = 1:2), and the organic solvent was removed using a rotary evaporator. The thin lipid film was then dissolved with UP water containing 5-FU by shaking in the water bath for 30 min and the suspension was ultrasonicated (300 W) for 5 min to retain homogeneous size. The TFS liquid was further centrifuged at 10,000 rpm for 10 min and then the unincorporated IMP and 5-FU were removed by filtration through a 0.22 μm cellulose nitrate membrane. Finally, TFS co-loaded with 5-FU and IMP was stored at 4 °C and labeled as 5-FU/IMP-TFS.

### 4.4. Optimization of TFS by BBD

Based on the scope to most efficiently relate the response variables and experimental results, BBD (Box-Behnken Design, Design-Expert 8.0.6) was used to optimize the preparation process of 5-FU/IMP-TFS and determine the suitable formulation. The independent variables include the drug/lipid ratio (*X*1), the SPC/TW-80 ratio (*X*2), and the hydration temperature (*X*3), with co-encapsulation efficiency (*Y*) as the experimental response variable.

### 4.5. Characterization of TFS

The particle size, polydispersity index (PDI), and zeta potential of the TFS were measured using a Malvern Zettaliter (Nano ZS, Malvern Panalytical Ltd., Malvern, UK) dynamic light scattering detector at room temperature. Before measurement, samples were diluted 1:10 (*v*/*v*) with UP water. The sample morphology was evaluated by TEM (HT7800, Hitachi, Tokyo, Japan). To verify the successful preparation of the TFS, its mid-infrared spectrum was measured using FT-IR (Clry610, Agilent, Santa Clara, CA, USA). The samples were prepared using the potassium bromide press method and the scanning wavelengths ranged from 4000 to 400 cm^−1^.

### 4.6. Encapsulation Efficiency (EE%) and Drug Loading (DL%)

The EE% and DL% of 5-FU/IMP-TFS were analyzed with the HPLC (Agilent 1260, Santa Clara, CA, USA). The samples were centrifuged for 20 min at 5000 rpm and 4 °C. Then, samples were subjected to column chromatography using 95:5 (*v*/*v*) water/methanol to yield 5-FU, and 30:70 (*v*/*v*) water/methanol to yield IMP. The analysis was completed using a C18 analytical column (4.6 mm × 150 mm, 5 µm), with a column temperature set at 30 °C. The injection volume was 10 µL, and the flow rate was 1.0 mL/min. The detection wavelengths were 265 nm and 300 nm, respectively. The EE% and DL% were calculated using the following equations:

$$EE\%=\frac{The\;weight\;of\;drug\;encapsulated}{The\;weight\;of\;the\;drug\;added}\times 100\%$$

$$DL\%=\frac{The\;weight\;of\;drug\;mearsured}{The\;weight\;of\;all\;the\;materials}\times 100\%$$

### 4.7. Drug Release, Skin Permeation, and Deposition Studies

The release profile of TFS was assessed by performing the cumulative in vitro studies in a water/ethanol medium (*v*/*v* = 70:30). TFS suspension was added to dialysis bags, and the samples were immersed in 40 mL of the release medium. The temperature was maintained at 37 ± 0.5 °C with a 100 rpm rotation rate. At predetermined time intervals (1, 2, 4, 6, 8, 10, 12, and 24 h), 1 mL of dissolution medium was withdrawn and replaced with an equal volume of fresh medium. The drug concentration was quantified using HPLC.

The ex vivo skin permeation was determined using a Franz diffusion cell. The cleaned skin was clamped between the donor and receptor compartments of the cell (0.64 cm^2^). The skin was mounted carefully with the stratum corneum facing the donor compartment, and the dermal side was exposed to the receptor medium. The skin in the donor compartment was treated with TFS gel and 5-FU cream and the receptor compartment (5 mL) was filled with a water/ethanol receptor medium (*v*/*v* = 70:30). The diffusion cell assembly was maintained at 32 ± 1 °C, with the contents of the receptor compartment stirred at 100 rpm using a magnetic stirrer. Samples were withdrawn at regular intervals (1, 2, 4, 6, 8, 10, 12, and 24 h) through the sampling port and replaced with an equal volume of fresh diffusion medium to maintain sink conditions throughout the experiment. Drug content was analyzed using HPLC. The experiment was conducted in triplicate.

To assess drug deposition in the skin, the mounted skin on the diffusion cell was carefully removed and washed with receptor medium. The skin was cut into small pieces and placed in a grinding tube. Methanol was added for tissue grinding, followed by ultrasonic treatment for 10 min. The mixture was then centrifuged at 10,000 rpm for 5 min, and the supernatant was filtered through a 0.22 μm cellulose nitrate membrane. The drug content of the samples was analyzed using the HPLC.

### 4.8. Preparation of TFS Gel

The TFS gel was prepared by incorporating the optimized TFS into the dispersion of Carbopol 940. A precise weight of 1% Carbopol 940 was slowly added to TFS suspension and stirred continuously (1000 rpm) at room temperature overnight until evenly dispersed. Triethanolamine was added dropwise with continuous stirring to achieve TFS gel.

### 4.9. Cell Culture

Cells in the logarithmic growth phase were seeded into 96-well plates at a density of 5 × 10^3^ cells per well in 10% FBS medium. After 24 h of incubation, the medium was replaced with 1% FBS medium containing different formulations for 48 h. Plates designated as controls were kept in the dark, while the others were irradiated with a UVA irradiator (365 nm) at a dose of 1.5 J/ cm^2^. After 12 h of incubation in 1% FBS medium, 10 µL of CCK-8 reagent was added, and the cells were further incubated for 1 h at 37 °C in the dark. Optical density was then measured at 450 nm using a microplate reader (Gen5, BioTek, Winooski, VT, USA). The experiments were performed in triplicate.

### 4.10. Animal Experiment Design

Female BALB/c-Nu mice (4–5 weeks, 18–22 g) were divided into 7 groups for different treatments. Cultured A375 cells were injected subcutaneously into each mouse at a concentration of 5 × 10^6^ cells. Antitumor tests were conducted with an equivalent tumor volume (around 150 mm^3^). Tumor volume was measured every three days and calculated as the following equation:

(2) $$Tumor\;volume=length\times {width}^{2}/2$$

The 7 groups were (*n* = 6): model, PDT, 5-FU-Cream, IMP-TFS-Gel, 5-FU/IMP-TFS-Gel, IMP-TFS-Gel Laser, and 5-FU/IMP-TFS-Gel Laser treated groups. The concentration of 5-FU or IMP in all preparations was 0.5% *w*/*w*. A spatula was used to apply the cream or gel formulation (200 mg) once daily for 21 consecutive days to the area (1 cm × 2 cm) where the tumor cells were injected. For PDT experiments, the residual drug was wiped off the administration site with a 75% alcohol cotton 4 h after application and administration. The site was then illuminated with UVA (6 J/cm^2^) daily. The inhibition rate of tumor growth (IRT) was calculated using the following formula: IRT = 100% × (mean tumor weight of the control group—mean tumor weight of the experimental group) / mean tumor weight of the control group. The mice were sacrificed 21 days after treatment, and their tumors were harvested and fixed in 10% neutral buffered formalin for further study. Histological analysis was performed using H&E staining, and tumors were sectioned into thin slices and processed routinely into paraffin.

### 4.11. Detection of the Generation of ROS

A375 cells were placed in 6-well plates at the density of 5 × 10^4^ cells per well for 24 h. After incubation with 5-FU/IMP-TFS (100 µM) for 4 h, cells were washed three times using a PBS buffer and added fresh medium. One plate was exposed to UVA (power dose = 1.5 J/cm^2^) for 10 min and another was kept in the dark incubation. After 12 h of incubation, the cells were washed and added with ROS probe 2′,7′-dichloroflurescin diacetate (DCFH-DA, Sigma-Aldrich, St. Louis, MO, USA). The cells were incubated for 30 min at 37 °C and the fluorescence intensity was measured using the flow cytometry with Green-B (GRN-B, 488 nm) fluorophores detection channel (Guava EasyCyteTM, Luminex, Austin, TX, USA).

### 4.12. Cell Cycle and Apoptosis Analysis

5 × 10^4^ A375 cells were seeded in 6-well plates for 24 h and treated with 5-FU/IMP-TFS (100 µM) for 4 h. The cells were washed three times using PBS buffer and fresh medium. One plate received UVA exposure (power dose = 1.5 J/cm^2^) for 10 min and another plate was incubated in the dark. The cells were harvested, washed, and fixed with ethanol at −20 °C for 20 h, and the suspension was centrifuged and washed twice with PBS (4 °C). Cells were stained with Guava cell cycle reagent according to the manufacturer’s instructions. Stained cells were analyzed by flow cytometry with Red-B fluorophores detection channel (Guava EasyCyteTM, Luminex, Austin, TX, USA).

Cell apoptosis was measured using the propidium iodide (PI) and Annexin V-FITC apoptosis assay kit. Cells were treated, harvested, and washed with PBS before being stained with PI/ Annexin V kit according to the manufacturer’s instructions. Stained cells were analyzed by flow cytometry with a Green-B, Red-B (488 nm) fluorophores detection channel (Guava EasyCyteTM, Luminex, Austin, TX, USA).

### 4.13. Statistical Analysis

All quantitative data are shown as mean ± SD, n ≥ 3. Statistical analyses were performed using GraphPad Prism 9.5.1 (MacOS) and Design-Expert 8.0.6 software and * *p* < 0.05, ** *p* < 0.01, *** *p* < 0.001, **** *p* < 0.0001.

## 5. Conclusions

In summary, we have developed a 5-FU/ IMP transdermal delivery system combined with chemo-photodynamic therapy for melanoma. The morphology of 5-FU/IMP-TFS was revealed with a particle size of 50 ± 1.92 nm and −13 ± 1.5 mV zeta potential. The in vitro drug release profiles and skin permeation studies were further evaluated. The steady-state rate of 5-FU in TFS was 1.47 times greater than conventional cream, and the retention was 4.43 times than that of the cream group. The study utilized 5-FU as the chemotherapeutic agent while IMP served as the photosensitizer, and 5-FU/IMP was co-encapsulated in the transdermal delivery vehicle TFS and embedded in Carbopol gel. The TFS gel has been shown to significantly improve skin permeability and increase local drug concentration compared to conventional creams. At the cellular level, the combination of 5-FU and IMP significantly reduced the toxicity of 5-FU in normal cells while enhancing the inhibition of tumor cells. Additionally, 5-FU/IMP-TFS, acting as both a chemotherapy and photodynamic agent, could improve anti-cancer efficiency when exposed to UVA laser sources. We also verified the results in nude mouse tumor models. The ROS generation, cell cycle, and cell apoptosis studies have proven the enhancement made by the combined chemo-photodynamic therapy. Overall, this study indicates that TFS, a transdermal delivery vehicle co-loaded with 5-FU and IMP, represents a novel therapeutic option for synergistic anti-cancer treatment.

## Figures and Tables

**Table 1:** The design of the factor levels.

Factors	Definition	Levels		
		−1	0	1
Drug/Lipid Ratio	*X*1	1:12.5	1:15.0	1:17.5
SPC/TW-80 Ratio	*X*2	6:4	7:3	8:2
Hydration Temp.	*X*3	35 °C	40 °C	45 °C

## Data Availability

The data are available upon request.

## Supplementary Materials

The following supporting information can be downloaded at: https://www.mdpi.com/article/10.3390/ijms25179611/s1.

## References

[1] Long G.V., Swetter S.M., Menzies A.M., Gershenwald J.E., Scolyer R.A. (2023). Cutaneous melanoma. Lancet.

[2] Rahman S., Haque T.N., Sugandhi V.V., Saraswat A.L., Xin X., Cho H. (2023). Topical Cream Carrying Drug-Loaded Nanogels for Melanoma Treatment. Pharm. Res..

[3] Siegel R.L., Giaquinto A.N., Jemal A. (2024). Cancer statistics, 2024. CA Cancer J. Clin..

[4] Eggermont A.M., Spatz A., Robert C. (2014). Cutaneous melanoma. Lancet.

[5] Chen K.G., Leapman R.D., Zhang G., Lai B., Valencia J.C., Cardarelli C.O., Vieira W.D., Hearing V.J., Gottesman M.M. (2009). Influence of melanosome dynamics on melanoma drug sensitivity. J. Natl. Cancer Inst..

[6] Fang D., Nguyen T.K., Leishear K., Finko R., Kulp A.N., Hotz S., Van Belle P.A., Xu X., Elder D.E., Herlyn M. (2005). A tumorigenic subpopulation with stem cell properties in melanomas. Cancer Res..

[7] Gottesman M.M. (2002). Mechanisms of cancer drug resistance. Annu. Rev. Med..

[8] Goette D.K. (1981). Topical chemotherapy with 5-fluorouracil. A review. J. Am. Acad. Dermatol..

[9] Safwat M.A., Soliman G.M., Sayed D., Attia M.A. (2018). Fluorouracil-Loaded Gold Nanoparticles for the Treatment of Skin Cancer: Development, in Vitro Characterization, and in Vivo Evaluation in a Mouse Skin Cancer Xenograft Model. Mol. Pharm..

[10] Crisóstomo L., Carvalho G.S.G., Leal L., de Araújo T.G., Nogueira K.A.B., da Silva D.A., de Oliveira Silva Ribeiro F., Petrilli R., Eloy J.O. (2022). Sorbitan Monolaurate-Containing Liposomes Enhance Skin Cancer Cell Cytotoxicity and in Association with Microneedling Increase the Skin Penetration of 5-Fluorouracil. AAPS PharmSciTech.

[11] Zhao H., Feng Y.L., Wang M., Wang J.J., Liu T., Yu J. (2022). The Angelica dahurica: A Review of Traditional Uses, Phytochemistry and Pharmacology. Front. Pharmacol..

[12] Nasser M.I., Zhu S., Hu H., Huang H., Guo M., Zhu P. (2019). Effects of imperatorin in the cardiovascular system and cancer. Biomed. Pharmacother..

[13] Kim T., Hyun C.G. (2022). Imperatorin Positively Regulates Melanogenesis through Signaling Pathways Involving PKA/CREB, ERK, AKT, and GSK3β/β-Catenin. Molecules.

[14] Wróblewska-Łuczka P., Grabarska A., Florek-Łuszczki M., Plewa Z., Łuszczki J.J. (2021). Synergy, Additivity, and Antagonism between Cisplatin and Selected Coumarins in Human Melanoma Cells. Int. J. Mol. Sci..

[15] Kimura Y., Sumiyoshi M., Sakanaka M., Taniguchi M., Baba K. (2013). In vitro and in vivo antiproliferative effect of a combination of ultraviolet-A and alkoxy furocoumarins isolated from Umbelliferae medicinal plants, in melanoma cells. Photochem. Photobiol..

[16] Guo Y., Liu H., Xiao H., Yuan M., Liu Y., Sedlařík V., Chin W.C., Liu J., Guo L., Li C. (2021). Self-assembled Camptothecin derivatives—Curcuminoids conjugate for combinatorial chemo-photodynamic therapy to enhance antitumor efficacy. J. Photochem. Photobiol. B.

[17] Dragicevic N., Predic-Atkinson J., Nikolic B., Pajovic S.B., Ivkovic S., Adzic M. (2024). Nanocarriers in topical photodynamic therapy. Expert Opin. Drug Deliv..

[18] Doppalapudi S., Jain A., Chopra D.K., Khan W. (2017). Psoralen loaded liposomal nanocarriers for improved skin penetration and efficacy of topical PUVA in psoriasis. Eur. J. Pharm. Sci..

[19] Keyal U., Bhatta A.K., Zhang G., Wang X.L. (2019). Present and future perspectives of photodynamic therapy for cutaneous squamous cell carcinoma. J. Am. Acad. Dermatol..

[20] Jain R., Paul M., Padaga S.G., Dubey S.K., Biswas S., Singhvi G. (2023). Dual-Drug-Loaded Topical Delivery of Photodynamically Active Lipid-Based Formulation for Combination Therapy of Cutaneous Melanoma. Mol. Pharm..

[21] Clemente N., Miletto I., Gianotti E., Sabbatini M., Invernizzi M., Marchese L., Dianzani U., Renò F. (2021). Verteporfin-Loaded Mesoporous Silica Nanoparticles’ Topical Applications Inhibit Mouse Melanoma Lymphangiogenesis and Micrometastasis In Vivo. Int. J. Mol. Sci..

[22] Champeau M., Vignoud S., Mortier L., Mordon S. (2019). Photodynamic therapy for skin cancer: How to enhance drug penetration?. J. Photochem. Photobiol. B.

[23] Benson H.A. (2006). Transfersomes for transdermal drug delivery. Expert Opin. Drug Deliv..

[24] Simrah Hafeez A., Usmani S.A., Izhar M.P. (2024). Transfersome, an ultra-deformable lipid-based drug nanocarrier: An updated review with therapeutic applications. Naunyn Schmiedebergs Arch. Pharmacol..

[25] Phatale V., Vaiphei K.K., Jha S., Patil D., Agrawal M., Alexander A. (2022). Overcoming skin barriers through advanced transdermal drug delivery approaches. J. Control. Release.

[26] Jiang T., Wang T., Li T., Ma Y., Shen S., He B., Mo R. (2018). Enhanced Transdermal Drug Delivery by Transfersome-Embedded Oligopeptide Hydrogel for Topical Chemotherapy of Melanoma. ACS Nano.

[27] Lin H., Xie Q., Huang X., Ban J., Wang B., Wei X., Chen Y., Lu Z. (2018). Increased skin permeation efficiency of imperatorin via charged ultradeformable lipid vesicles for transdermal delivery. Int. J. Nanomed..

[28] Souto E.B., Macedo A.S., Dias-Ferreira J., Cano A., Zielińska A., Matos C.M. (2021). Elastic and Ultradeformable Liposomes for Transdermal Delivery of Active Pharmaceutical Ingredients (APIs). Int. J. Mol. Sci..

[29] Cosco D., Paolino D., Maiuolo J., Marzio L.D., Carafa M., Ventura C.A., Fresta M. (2015). Ultradeformable liposomes as multidrug carrier of resveratrol and 5-fluorouracil for their topical delivery. Int. J. Pharm..

[30] Demartis S., Rassu G., Murgia S., Casula L., Giunchedi P., Gavini E. (2021). Improving Dermal Delivery of Rose Bengal by Deformable Lipid Nanovesicles for Topical Treatment of Melanoma. Mol. Pharm..

[31] Verma D.D., Verma S., Blume G., Fahr A. (2003). Particle size of liposomes influences dermal delivery of substances into skin. Int. J. Pharm..

[32] Hussain A., Haque M.W., Singh S.K., Ahmed F.J. (2016). Optimized permeation enhancer for topical delivery of 5-fluorouracil-loaded elastic liposome using Design Expert: Part II. Drug Deliv..

[33] Chen T., Chen H., Jiang Y., Yan Q., Zheng S., Wu M. (2022). Co-Delivery of 5-Fluorouracil and Paclitaxel in Mitochondria-Targeted KLA-Modified Liposomes to Improve Triple-Negative Breast Cancer Treatment. Pharmaceuticals.

[34] Wang J., Zhao Y., Zhai B., Cheng J., Sun J., Zhang X., Guo D. (2023). Phloretin Transfersomes for Transdermal Delivery: Design, Optimization, and In Vivo Evaluation. Molecules.

[35] Yuan M., Niu J., Xiao Q., Ya H., Zhang Y., Fan Y., Li L., Li X. (2022). Hyaluronan-modified transfersomes based hydrogel for enhanced transdermal delivery of indomethacin. Drug Deliv..

[36] Cosco D., Celia C., Cilurzo F., Trapasso E., Paolino D. (2008). Colloidal carriers for the enhanced delivery through the skin. Expert Opin. Drug Deliv..

[37] Hussain A., Samad A., Ramzan M., Ahsan M.N., Ur Rehman Z., Ahmad F.J. (2016). Elastic liposome-based gel for topical delivery of 5-fluorouracil: In vitro and in vivo investigation. Drug Deliv..

[38] Cadet J., Douki T. (2018). Formation of UV-induced DNA damage contributing to skin cancer development. Photochem. Photobiol. Sci..

[39] Gag O., Dinu Ș., Manea H., Marcovici I., Pînzaru I., Popovici R., Crăiniceanu Z., Gyori Z., Iovănescu G., Chiriac S. (2023). UVA/UVB Irradiation Exerts a Distinct Phototoxic Effect on Human Keratinocytes Compared to Human Malignant Melanoma Cells. Life.

[40] Marrelli M., Giordano F., Perri M.R., Amodeo V., Baldino N., Lupia C., Uzunov D., Musolino V., Conforti F., Panno M.L. (2023). Phytochemical Profile and In Vitro Antioxidant and Photobiological Properties of Different Extracts from Prangos ferulacea Lindl. Antioxidants.

[41] Marrelli M., Perri M.R., Amodeo V., Giordano F., Statti G.A., Panno M.L., Conforti F. (2021). Assessment of Photo-Induced Cytotoxic Activity of Cachrys sicula and Cachrys libanotis Enriched-Coumarin Extracts against Human Melanoma Cells. Plants.

[42] Rapa S.F., Magliocca G., Pepe G., Amodio G., Autore G., Campiglia P., Marzocco S. (2021). Protective Effect of Pomegranate on Oxidative Stress and Inflammatory Response Induced by 5-Fluorouracil in Human Keratinocytes. Antioxidants.

[43] Deng M., Xie L., Zhong L., Liao Y., Liu L., Li X. (2020). Imperatorin: A review of its pharmacology, toxicity and pharmacokinetics. Eur. J. Pharmacol..

[44] Huang Y., Lai H., Jiang J., Xu X., Zeng Z., Ren L., Liu Q., Chen M., Zhang T., Ding X. (2022). pH-activatable oxidative stress amplifying dissolving microneedles for combined chemo-photodynamic therapy of melanoma. Asian J. Pharm. Sci..

[45] Huang L., Chen X., Bian Q., Zhang F., Wu H., Wang H., Gao J. (2020). Photosensitizer-stabilized self-assembling nanoparticles potentiate chemo/photodynamic efficacy of patient-derived melanoma. J. Control. Release.

[46] Xu S., Liu C., Zang S., Li J., Wang Y., Ren K., Li M., Zhang Z., He Q. (2021). Multifunctional self-delivery micelles targeting the invasion-metastasis cascade for enhanced chemotherapy against melanoma and the lung metastasis. Asian J. Pharm. Sci..

[47] Gilani S.J., Jahangir M.A., Chandrakala, Rizwanullah M., Taleuzzaman M., Shahab M.S., Shakeel K., Aqil M., Imam S.S. (2018). Nano-Based Therapy for Treatment of Skin Cancer. Recent Pat. Anti-Infect. Drug Discov..

[48] Moller P.L., Sindet-Pedersen S., Petersen C.T., Juhl G.I., Dillenschneider A., Skoglund L.A. (2005). Onset of acetaminophen analgesia: Comparison of oral and intravenous routes after third molar surgery. Br. J. Anaesth..

[49] Li H., Wang Y., Tang Q., Yin D., Tang C., He E., Zou L., Peng Q. (2021). The protein corona and its effects on nanoparticle-based drug delivery systems. Acta Biomater..

[50] Wen W., Wu J., Liu L., Tian Y., Buettner R., Hsieh M.Y., Horne D., Dellinger T.H., Han E.S., Jove R. (2015). Synergistic antitumor effect of combined inhibition of EGFR and JAK/STAT3 pathways in human ovarian cancer. Mol. Cancer.

[51] Akbar A., Khan S., Chatterjee T., Ghosh M. (2023). Unleashing the power of porphyrin photosensitizers: Illuminating breakthroughs in photodynamic therapy. J. Photochem. Photobiol. B.

[52] Gaona-Esquivel A., Diana S.H.-M., Hernández-Rodríguez Y.M., Cigarroa-Mayorga O.E. (2022). The role of Nd as a dopant in Mn3O4NPs on the heat induction of artificial breast tissue due to the irradiation of microwaves. Mater. Chem. Phys..

[53] Simo L.M., Messi L.M., Mbing J.N., Muller C.D., Boyom F.F., Begoudé A.B., Pegnyemb D.E., Haddad M., Noté O.P. (2023). A New Triterpenoid Saponin from Albizia zygia Induced Apoptosis by Reduction of Mitochondrial Potential Status in Malignant Melanoma Cells. Planta Med..

[54] Xu J., Sun Y., Huang J., Chen C., Liu G., Jiang Y., Zhao Y., Jiang Z. (2007). Photokilling cancer cells using highly cell-specific antibody-TiO(2) bioconjugates and electroporation. Bioelectrochemistry.

[55] Nackiewicz J., Kliber-Jasik M., Pogoda-Mieszczak K., Skonieczna M. (2024). Gallium octacarboxyphthalocyanine hydroxide as a potential pro-apoptotic drug against cancer skin cells. Bioorg. Chem..

