# Supplementary material for: Topical Delivery of Dual Loaded Nano-Transfersomes Mediated Chemo-Photodynamic Therapy against Melanoma via Inducing Cell Cycle Arrest and Apoptosis
Source: Int J Mol Sci. 2024 Sep 5;25(17):9611. doi: 10.3390/ijms25179611 (PMC11394987; doi:10.3390/ijms25179611)
Supplement: Supplementary file 1 [file ijms-25-09611-s001.zip › ijms-3143607-supplementary.pdf]

Table S1. Optimizing TFS using BBD (n=3, mean ± SD).

Formulation		Factors			Responses		
Std	Run	X1	X2	X3	EE_{IMP} (%)	EE_{FU} (%)	Y (%)
1	1	1:12.5	6:4	40 °C	84.69 ± 3.6	73.72 ± 2.7	79.21
12	2	1:15.0	8:2	45 °C	86.49 ± 2.5	76.57 ± 1.4	81.53
3	3	1:12.5	8:2	40 °C	85.26 ± 3.7	76.24 ± 3.1	80.75
17	4	1:15.0	7:3	40 °C	89.94 ± 4.4	81.74 ± 2.9	85.84
4	5	1:17.5	8:2	40 °C	84.10 ± 1.8	75.87 ± 3.7	79.99
8	6	1:17.5	7:3	45 °C	85.88 ± 0.9	76.41 ± 2.2	81.14
11	7	1:15.0	6:4	45 °C	83.75 ± 7.1	75.94 ± 2.2	79.84
7	8	1:12.5	7:3	45 °C	87.71 ± 6.3	77.35 ± 2.2	82.53
9	9	1:15.0	6:4	35 °C	83.21 ± 3.0	70.55 ± 2.9	76.88
14	10	1:15.0	7:3	40 °C	88.79 ± 2.9	79.98 ± 2.6	84.39
16	11	1:15.0	7:3	40 °C	89.73 ± 2.7	80.58 ± 0.4	85.15
15	12	1:15.0	7:3	40 °C	89.30 ± 1.6	80.21 ± 3.6	84.75
13	13	1:15.0	7:3	40 °C	87.82 ± 1.2	79.50 ± 5.7	83.66
6	14	1:17.5	7:3	35 °C	83.19 ± 6.4	70.80 ± 3.8	76.99
5	15	1:12.5	7:3	35 °C	81.41 ± 7.6	72.83 ± 2.4	77.12
2	16	1:17.5	6:4	40 °C	84.64 ± 2.2	73.60 ± 2.9	79.12
10	17	1:15.0	8:2	35 °C	80.67 ± 8.5	71.96 ± 5.6	76.31

Table S2. ANOVA for Response Surface Quadratic Model.

Source	Squares	*df*	Mean Square	*F*-Value	*P*-Value
Model	0.015	9	$1.686*10^{-3}$	37.20	<0.0001
X_1	$7.011*10^{-5}$	1	$7.011*10^{-5}$	1.55	0.2537
X_2	$1.557*10^{-4}$	1	$1.557*10^{-4}$	3.43	0.1063
X_3	$3.929*10^{-3}$	1	$3.929*10^{-3}$	86.66	<0.0001
X_1X_2	$1.159*10^{-5}$	1	$1.159*10^{-5}$	0.26	0.6286
X_1X_2	$3.950*10^{-5}$	1	$3.950*10^{-5}$	0.87	0.3817
X_2X_3	$1.265*10^{-4}$	1	$1.265*10^{-4}$	2.79	0.1388
X_1^2	$1.846*10^{-3}$	1	$1.846*10^{-3}$	40.71	0.0004
X_2^2	$3.539*10^{-3}$	1	$3.539*10^{-3}$	78.05	<0.0001
X_3^2	$4.362*10^{-3}$	1	$4.362*10^{-3}$	96.20	<0.0001
Residual	$3.174*10^{-4}$	7	$4.534*10^{-5}$		
Lack of Fit	$5.040*10^{-5}$	3	$1.680*10^{-5}$	0.25	0.8569
Pure Error	$2.670*10^{-4}$	4	$6.674*10^{-5}$		
Cor Total	0.015	16	$1.686*10^{-3}$		

Table S3. Encapsulation Rate of TFS

Number	EE_{IMP}	EE_{FU}	$EE_{5\text{-}FU/IMP}$	$EE_{5\text{-}FU/IMP}$ (projected value)	*Absolute deviation*	*RSD*
1	88.49%	80.23%	84.36%		0.99%	
2	88.80%	79.92%	84.36%	85.20%	0.99%	0.9%
3	92.38%	78.98%	85.68%		0.56%	

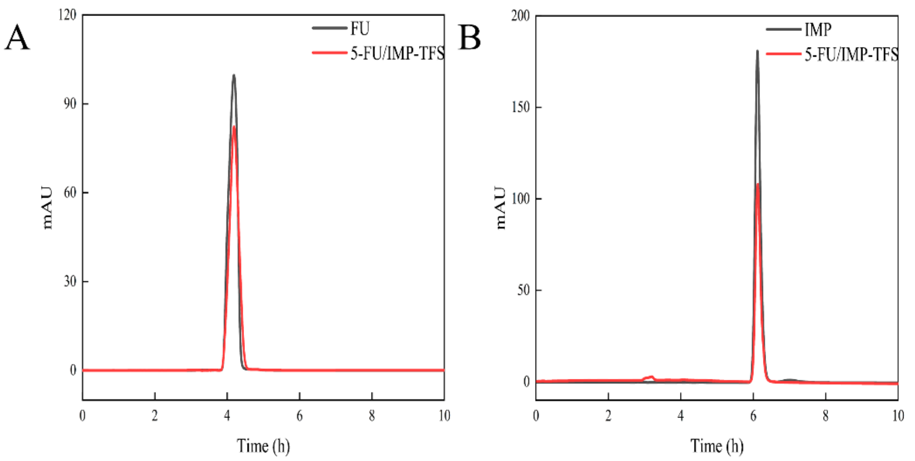

Fig. S1. The HPLC Chromatograms. (A) 5-FU, (B) IMP.

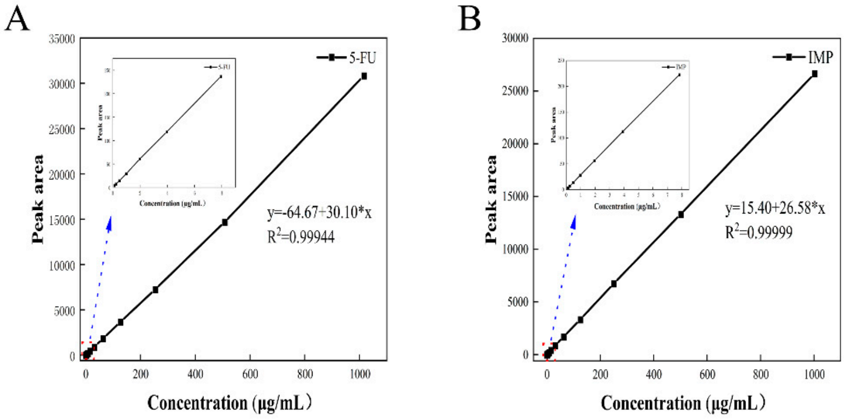

Fig. S2. The HPLC Standard Curves. (A) 5-FU, (B) IMP.

Table S4. HPLC Precision Test

	Concentration (μg)	Peak area						Average peak area	RSD (%)
		1	2	3	4	5	6		
IMP	125.63	3212.6	3207.6	3205.4	3205.7	3205.3	3206.4	3207.17±2.55	0.08
	251.25	6498.5	6501.1	6489.0	6496.8	6495.5	6498.6	6496.58±3.81	0.06
	502.50	12666.2	12667.2	12646.9	12661.0	12678.3	12653.0	12662.10±10.17	0.08
FU	132.75	4028.5	4031.7	4033.5	4029.7	4033.3	4034.4	4031.85±2.13	0.05
	265.50	7998.9	8114.6	8112.8	8121.4	8126.7	8080.1	8092.42±44.38	0.55
	531.00	16709.0	16731.6	16704.2	16720.8	16494.2	16524.1	16647.32±98.46	0.59

Table S5. HPLC Stability Test

	Concentration (μg)	Peak area						Average peak area	RSD (%)
		0 h	3 h	6 h	12 h	18 h	24 h		
IMP	62.81	1627.5	1609.4	1615.9	1612.4	1646.4	1616.2	1621.30±12.55	0.77
FU	66.38	1970.5	1975.5	1940.6	1930.8	1970.2	1975.3	1960.48±17.87	0.91

Table S6. HPLC Repeatability Test

	Concentration (μg)	Peak area						Average peak area	RSD (%)
		1	2	3	4	5	6		
IMP	62.81	1616.5	1617.4	1614.0	1613.1	1612.1	1611.4	1614.08±2.20	0.14
FU	66.38	1963.5	1970.4	1968.6	1965.9	1973.0	1970.7	1968.68±3.17	0.16

Table S7. IMP Sample HPLC Recovery Rate

	Preparations	Concentration (μg)	Preparation peak area	Control peak area	Recovery rate (%)	Average recovery rate (%)	RSD (%)
IMP	TFS	255.50	659.7	655.1	100.71	99.37±1.28	1.29
			653.4		99.75		
			639.6		97.64		
		127.75	325.8	338.5	96.25	96.16±0.74	0.77
			322.3		95.21		
			328.4		97.01		
		63.88	161.1	167.1	96.42	97.27±1.43	1.47
			160.6		96.12		
			165.9		99.29		
	TFS gel	251.25	613.5	632.3	97.02	99.96±2.13	2.13
			645.0		102.00		
			637.7		100.85		
		125.63	313.5	324.5	96.63	96.58±1.08	1.12
			317.6		97.89		
			309.0		95.24		
		62.81	159.5	160.4	99.46	99.11±0.31	0.31
			158.3		98.72		
			159.0		99.15		

Table S8. 5-FU Sample HPLC Recovery Rate

	Preparations	Concentration (μg)	Preparation peak area	Control peak area	Recovery rate (%)	Average recovery rate (%)	RSD (%)
FU	TFS	259.00	775.4	789.5	98.21	98.92±0.58	0.59
			781.0		98.92		
			786.6		99.63		
		129.50	381.0	390.6	97.53	97.71±0.45	0.46
			380.0		97.28		
			384.1		98.33		
		64.75	192.3	189.2	101.63	99.16±1.95	1.96
			183.3		96.87		
			187.3		98.99		
	TFS gel	251.25	801.3	824.7	97.16	100.49±2.53	2.52
			851.8		103.28		
			833.2		101.03		
		125.63	386.1	399.9	96.54	96.81±0.54	0.55
			390.2		97.56		
			385.3		96.34		
		62.81	209.0	201.4	103.76	102.21±1.14	1.11
			205.0		101.77		
			203.6		101.08		
